# Cucurbit [8] uril-based supramolecular fluorescent biomaterials for cytotoxicity and imaging studies of kidney cells

**DOI:** 10.3389/fchem.2022.974607

**Published:** 2022-08-24

**Authors:** Han Xiao, Xia Yang, Li Yang, Dan Yang, Yang Luo, Hai-Ping Yang, Zhu Tao, Xin Xiao, Qiu Li

**Affiliations:** ^1^ Department of Nephrology, Children’s Hospital of Chongqing Medical University, National Clinical Research Center for Child Health and Disorders, Ministry of Education Key Laboratory of Child Development and Disorders, Chongqing Key Laboratory of Pediatrics, Chongqing, China; ^2^ Key Laboratory of Macrocyclic and Supramolecular Chemistry of Guizhou Province, Institute of Applied Chemistry, Guizhou University, Guiyang, China

**Keywords:** acute kidney injury, supramolecular fluorescent biomaterials, excellent fluorescence properties, low biological toxicity, macrocyclic host

## Abstract

An accurate diagnosis of acute kidney injury (AKI) at the early stage is critical to not only allow preventative treatments in time but also forecast probable medication toxicity for preventing AKI from starting and progressing to severe kidney damage and death. Therefore, supramolecular fluorescent biomaterials based on Q [8] and PEG-APTS have been prepared herein. This study has found that the unique properties of outer surface methine and the positive density of Q [8] can form a stable assembly with PEG-APTS, and has provided the possibility for the faster crossing of the glomerular filtration barrier to enter into the resident cells of the kidney. In addition to the excellent fluorescence properties, the as-synthesized biomaterial Q [8]@PEG-APTS has possessed significantly low biological toxicity. Most importantly, the accumulation of Q [8]@PEG-APTS in large amounts in cytoplasm and nucleus of HK2 and HMCs cells, respectively, within 24 h enabled distinguishing kidney cells when diagnosing and providing some foundation for early AKI.

## Graphical Abstract

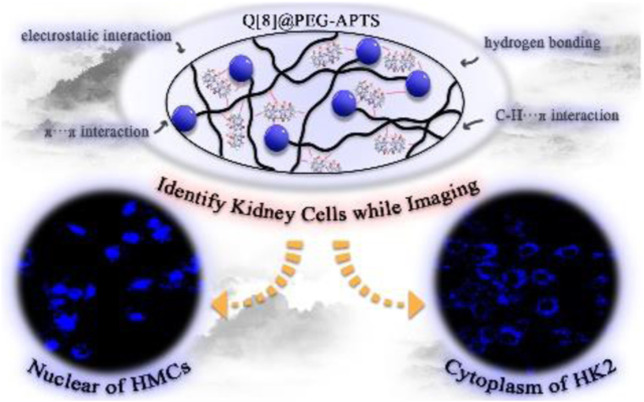

## Introduction

Acute kidney injury (AKI) is broadly defined as the sudden loss of kidney function and has become a worldwide health problem to expedite morbidity and mortality (Chawla et al., 2014; Kellum et al., 2021). Several conditions can precipitate AKI (Goldstein et al., 2016; Sato et al., 2017; Kao et al., 2019; Peerapornratana et al., 2019), such as sepsis, hypotension, organ failure, kidney stone, and abusing medications. Moreover, variegated medications, such as cisplatin (anticancer drug), acyclovir (antiviral agent), aminoglycoside (antibiotic), and others, have been proven to cause severe nephrotoxicity and AKI in people (Pazhayattil and Shirali, 2014). The accurate diagnosis of AKI at an early stage is critical in not only offering preventative treatments in time, but also forecasting probable medication toxicity for preventing AKI from starting, spreading, and propagating into severe kidney damage and death (Basile et al., 2016). Traditional imaging methods, (Lerman et al., 1999; Kobayashi et al., 2002; Fisch et al., 2016) such as computed tomography, magnetic resonance imaging, and ultrasound imaging, help for AIK diagnosis, primarily through noninvasive *in vivo* imaging of adjustments in kidney anatomy and histology, yet, the lack of capacity to detect AKI-associated biomarkers at the molecular level has hampered the capacity to discern AKI at an early stage. Recently, fluorescence imaging (Cui et al., 2020; Moore et al., 2021; Wu et al., 2021) has shown potential for AKI diagnosis because of its high sensitivity, low cost, and simplicity to use. It has the potential to offer valuable information on kidney anatomic changes, decreased renal perfusion, and aberrant biomolecule levels in the kidney, allowing noninvasive and real-time diagnosis of kidney dysfunction in patients.

In recent years, researchers have become much more interested in supramolecular self-assemblies incorporating fluorescence for cell imaging (Palivan et al., 2016; Li et al., 2018). Few non-fluorescent organic dyes can be transformed into fluorescent to emit diverse fluorescence emissions in supramolecular self-assemblies *via* either host-guest complexation or triggering the aggregation-induced emission (AIE) effect (Mei et al., 2015). At high concentrations, supramolecular self-assemblies may also inhibit dye aggregation and aggregation-induced quenching (ACQ). Therefore, a large number of supramolecular self-assembled materials have been applied in cellular imaging or other smart materials. Cucurbit [*n*]uril [Q (*n*)s or CB (*n*)s, *n* = 5–8, 10, 13–15], is usually used for designing supramolecular assemblies (Kim et al., 2000; Cheng et al., 2013; Chen et al., 2021; Liu et al., 2021; Luo et al., 2022a; Zhang et al., 2022a; Luo et al., 2022b; Zhang et al., 2022b; Zhang et al., 2022c). Cucurbit [n] urils are classical macrocyclic hosts formed by acid-catalyzed condensation of glycoluril and formaldehyde (Figure 2) having cavity sizes within 2.4–11.0 Å and a common depth of 9.1 Å. From the structural point of view, the rigid cavity of Q [*n*]s derives the classical Q [*n*]-based host-guest chemistry, the rich carbonyl oxygen of its portal yields the Q [*n*]-based coordination chemistry, and positively density outer surface of the Q [*n*] rich in methane breeds the novel outer surface interaction of Q [*n*] (Ni et al., 2014). Therefore, compared with other macrocycles (Liu Z. et al., 2017; Murray et al., 2017), Q [*n*]s distinguish themselves *via* the excellent ability to form inclusion complexes with various guest molecules through host-guest, coordination, and out surface interactions (Assaf and Nau, 2015; Barrow et al., 2015; Liu J. et al., 2017; Jia et al., 2019; Lin et al., 2020; Yang et al., 2021; Zhang et al., 2021; Wang et al., 2022).

**SCHEME 1:**
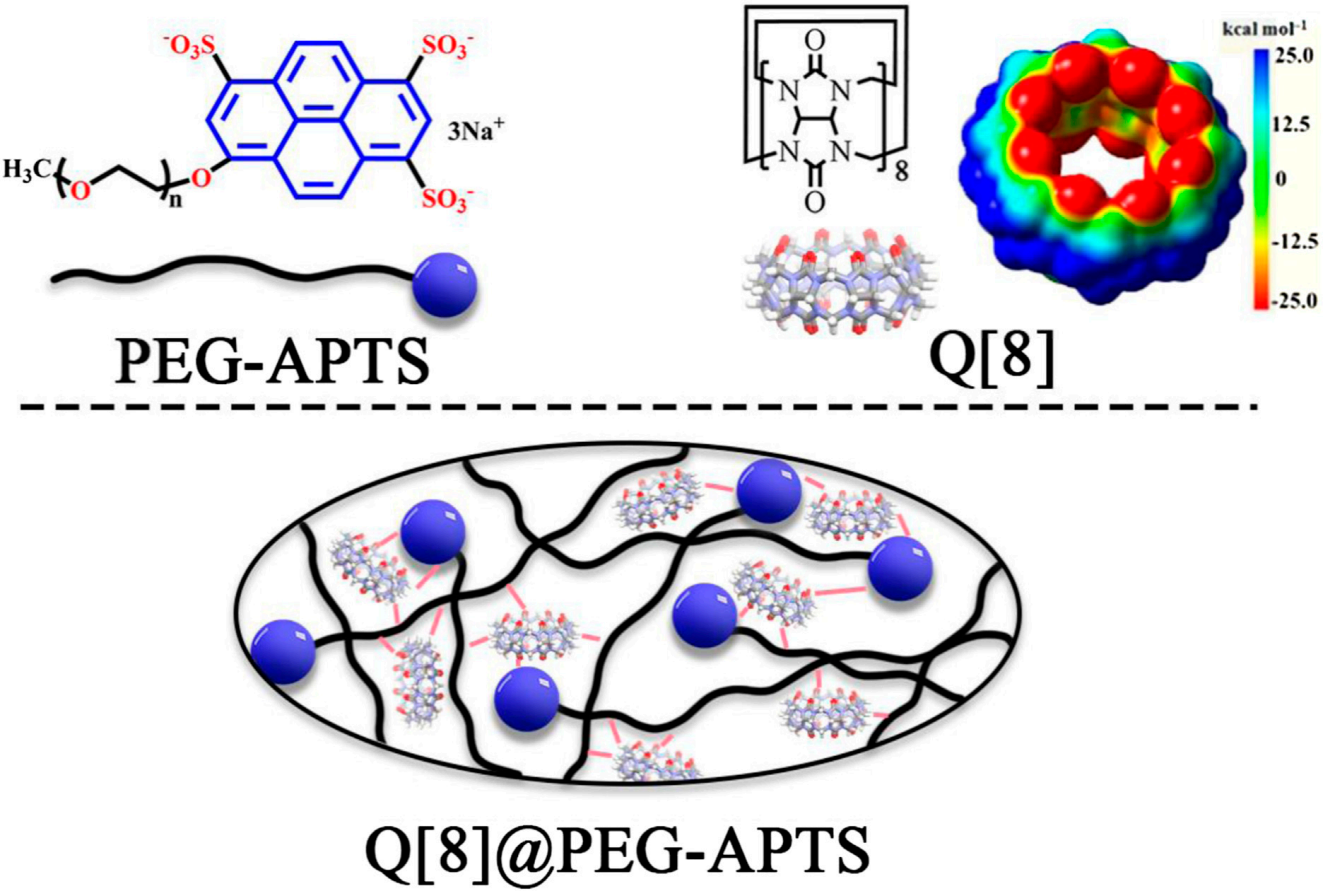
The structures of Q [8], PEG-APTS, and Q [8]@PEG-APTS.

Herein, a polymer chain (i.e., PEG-APTS, Scheme 1 for structure, Supplementary Scheme S2 for synthetic route) was designed by utilizing polyethylene glycol and sodium pyrene sulfonate. Q [8] was selected as the supramolecular host because of its excellent cavity properties among other common cucurbit [*n*] urils. Subsequently, the supramolecular fluorescent biomaterial Q [8]@PEG-APTS, synthesize by employing the outer surface interaction within PEG-APTS and Q [8], was demonstrated to possess a stable and uniform structure and good fluorescence properties. Most importantly, the positive density on the outer surface of Q [8] could neutralize the negative charge of sulfonate on PEG-APTS, enabling the rapid infiltration of Q [8]@PEG-APTS through the glomerular filtration barrier and entering the kidney resident cells. In addition, the as-synthesized biomaterial also possessed low biotoxicity and is capable of massive intracellular accumulation within 24 h. Moreover, Q [8]@PEG-APTS was found to track specifically different organelles in different cells, such as the selective accumulation in the cytoplasm in HK2 cells, specifically in the perinuclear of HMCs cells, which might be helpful to identify diverse cell types during tracing living cells in the kidney. This work provides an ideal cellular imaging supramolecular fluorescent biomaterial for the early-stage diagnosis of AKI and provides a theoretical basis for the potential application of Q [*n*]-based fluorescent biomaterials in kidney research and therapy.

## Results and discussion

### Preparation and characterization of Q [8]@PEG-APTS

**FIGURE 1:**
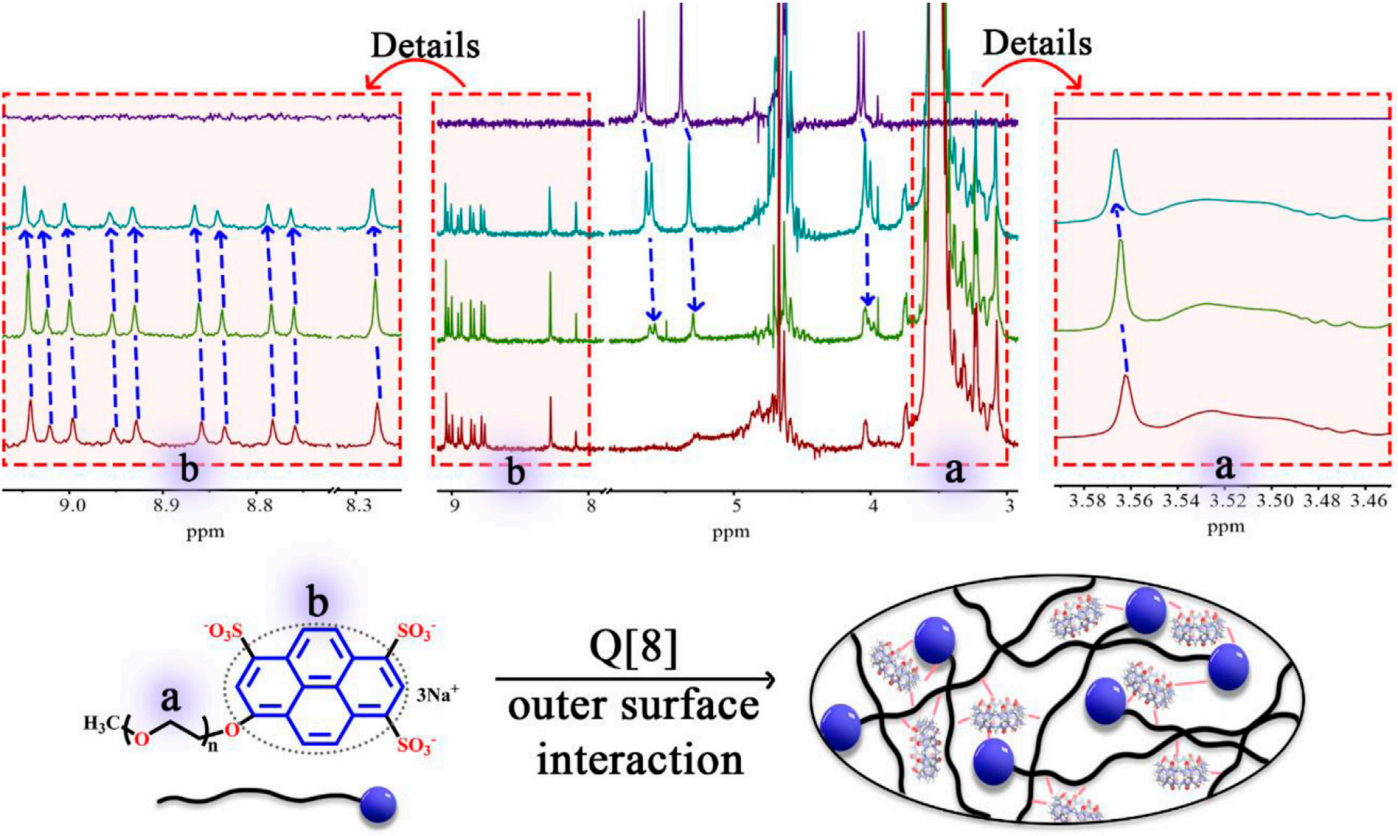
^1^H NMR spectrum (400 MHz, D_2_O) of PEG-APTS (dark red) with an increasing amount of Q [8] (green for host/guest ratio of 1:1, and dark green for host/guest ratio of 4.5:1), and Q [8] (dark blue).

Based on the abundant hydrogen bonding sites, such as–SO_3_H functionalities and oxygen atoms, and the negative charge on PEG-APTS, fabrication of the stable supramolecular fluorescent materials can be achieved from PEG-APTS through the outer surface interaction of Q [8]. ^1^H NMR titration, one of the most important tools to study supramolecular assembly, was carried out. From Figure 1, intensities of ^1^H NMR peaks of both alkoxy chain and pyrene group of PEG-APTS shifted downfield after the addition of Q [8], which inferred the interaction of these protons with the deshielding effect of the outer surface of Q [8]. On contrary, the proton signal of Q [8] appeared at the more upfield position because of the altered microenvironment of the outer surface. This series of changes in the proton signal revealed powerful interactions within PEG-APTS and Q [8]. These interactions arrived from the abundant hydrogen bonding between oxygen atom in the alkoxy chain and the exposed methine on the outer surface of Q [8], C-H···π interaction of pyrene molecule with methine, π···π interaction of pyrene and carbonyl oxygen, and electrostatic interaction within–SO_3_H and the outer surface of Q [8]. Such rich interactions greatly improved the stability of supramolecular fluorescent materials, thereby providing the basis for subsequent studies.

**FIGURE 2:**
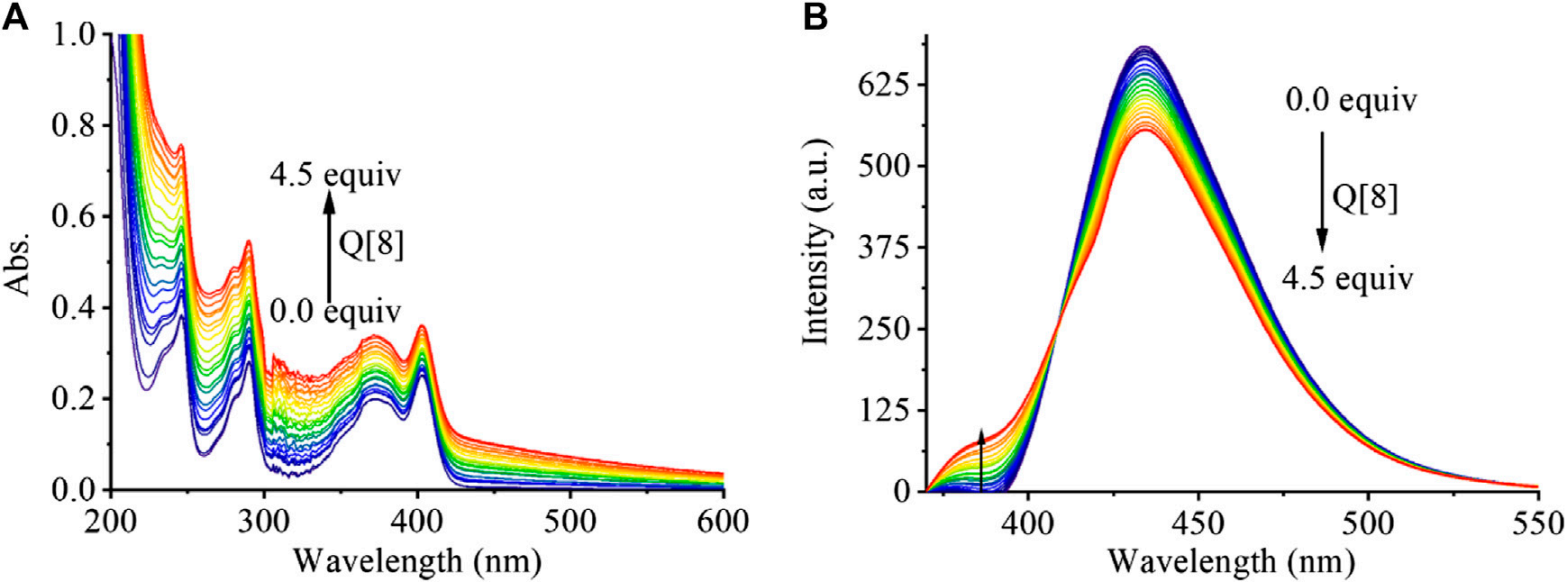
The UV-vis spectra **(A)** and fluorescence emission spectra **(B)** of PEG-APTS (20 μM) in the presence of an increasing amount of Q [8] from 0 … to 4.5 equivalents.

In addition to ^1^H NMR spectroscopy, both UV and fluorescence titration, the most common methods used understanding for supramolecular assemblies, were studied. As shown in Figure 2A, the absorption of PEG-APTS gradually increased with increasing Q [8] concentration because that the outer surface interaction of Q [8] and PEG-APTS may induce the strong *n*-π* and π-π* interactions. From the fluorescence titration, it was also observed that the addition of Q [8] slightly weakened the fluorescence performance of PEG-APTS, which indicated the elevated ACQ effect of fluorescent pyrene because the outer surface interaction, thereby improving the performance of supramolecular fluorescent materials disguisedly. To further characterize the morphology and size of supramolecular fluorescent material, DLS and SEM analyses were carried out.

From DLS results (Supplementary Figure S4), particle sizes of Q [8] (7.63 nm) and PEG-APTS (65.46 nm) were found to be relatively smaller than that of Q [8]@PEG-APTS (1149.93 nm), inferring the formation of Q [8]@PEG-APTS. From the SEM photomicrograph of Q [8]@PEG-APTS (Supplementary Figure S5), the supramolecular fluorescent material was observed to contain a regular, flattened ellipsoid surface.

### Cytotoxicity studies of Q [8]@PEG-APTS in cells

**FIGURE 3:**
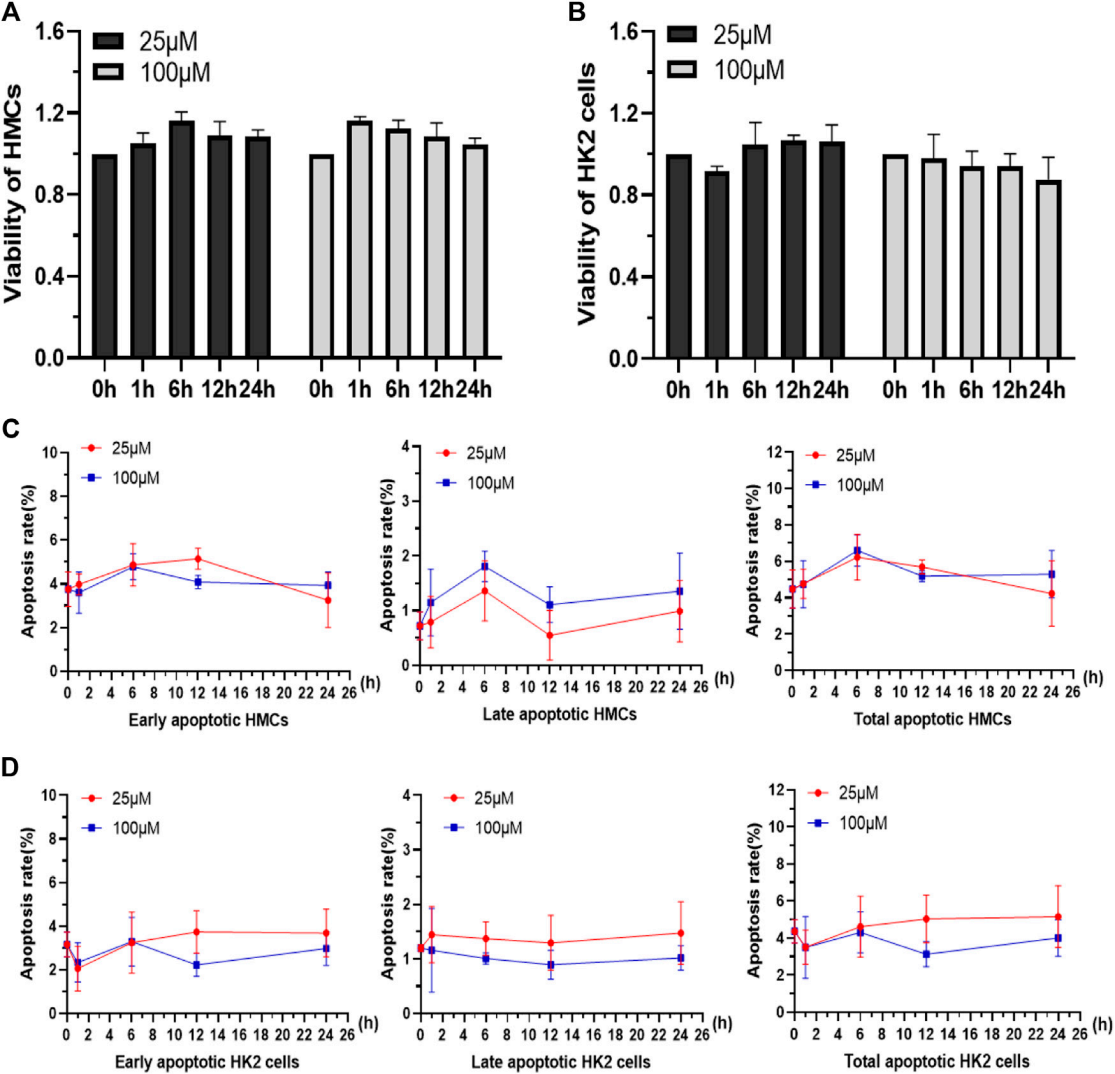
Viability of HMCs **(A)** and HK2 cells **(B)** incubated with 25 and 100 μM Q [8]@PEG-APTS for 0, 1, 6, 12 and 24 h. Apoptosis rates of HMCs **(C)** and HK2 cells **(D)** incubated with 25 and 100 μM Q [8]@PEG-APTS for 0, 1, 6, 12 and 24 h.

Because of the aforementioned properties, the as-synthesized macromolecule was investigated to be used as a tool for kidney live cell tracing and drug detection *in vivo*. Herein, some preliminary works were carried out to investigate the application of Q [8]@PEG-APTS for tracing kidney live resident cells, such as HMC and HK2. The kidney is considered one of the most important metabolically active organs that consisted of several resident cells, such as mesangial cells, proximal tubular epithelial cells (PTECs), podocytes, and many more. Of these, mesangial cells provide structural support for capillary loops and produce components of the glomerular matrix, (Kikkawa et al., 2003; Schlöndorff and Banas, 2009; Shotorbani et al., 2020; Avraham et al., 2021) whereas PTECs regulate the acid-base balance and body fluids homeostasis through reabsorption and secretion of electrolytes/metabolites (Nigam et al., 2015). From Figures 3A,B, HMCs and HK2 cells treated with 25 μM or 100 μM Q [8]@PEG-APTS exhibited no significant differences in relative viabilities among 0, 1, 6, 12, and 24 h groups. It was found that the viability rates of HMCs and HK2 cells cultured with Q [8]@PEG-APTS reached up to 85% under prolonged exposure to high concentrations (100 μM, 24 h). In addition, the early-, late-, and total-stage cellular apoptosis rates were not statistically different from the stimulation group, in which culture time was even up to 24 h for both cells. This has inferred that Q [8]@PEG-APTS possess low cytotoxicity towards HMCs and HK2 cells and therefore, can further be applied for cell imaging and *in vivo* studies (Figures 3C,D).

### Fluorescence imaging of Q [8]@PEG-APTS in cells

**FIGURE 4:**
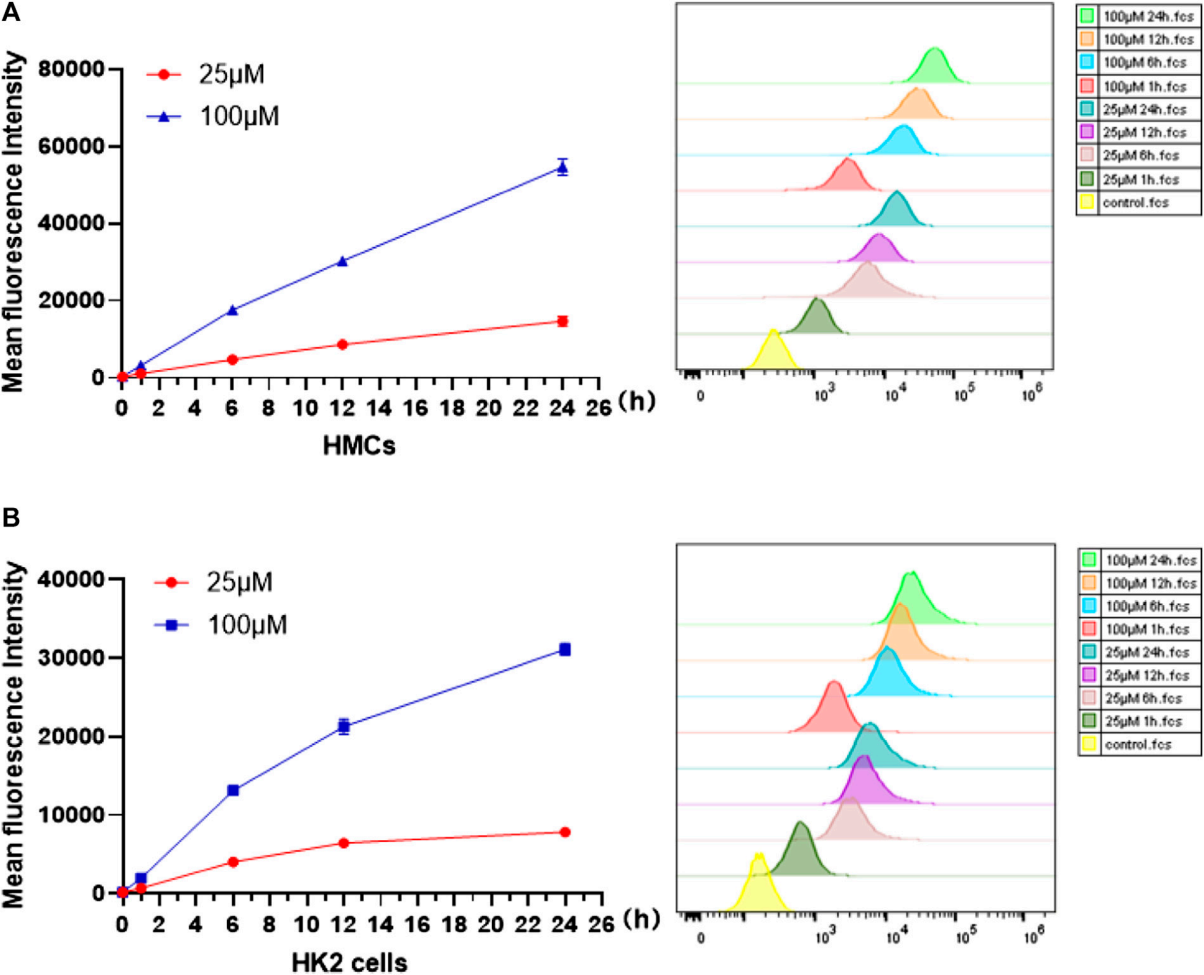
Mean fluorescence intensity of HMCs **(A)** and HK2 cells**(B)** incubated with 25 and 100 μM Q [8]@PEG-APTS for 0, 1, 6, 12 and 24 h.

Before cell imaging, changes in fluorescence intensity in presence of Q [8]@PEG-APTS assemblies were investigated initially by using cell flowmetry. From Figure 4A,B, the mean fluorescence intensity (MIF) of Q [8]@PEG-APTS absorbed by HMCs and HK2 cells was found to increase gradually with the increase in incubation time and concentration to reach the maxima at 24 h with 100 μM. Moreover, the significantly increased fluorescence signals of Q [8]@PEG-APTS were observed in cells incubated with 25 μM for 1 h compared to the control group (Figure 4A,B). The above results indicate that Q [8]@PEG-APTS assemblies were able to accumulate within the kidney cells in a relatively short period and reach the maxima at 24 h, possessing a very good kidney cell filtration capacity and the ability to be stable in cells and accumulate continuously.

**FIGURE 5:**
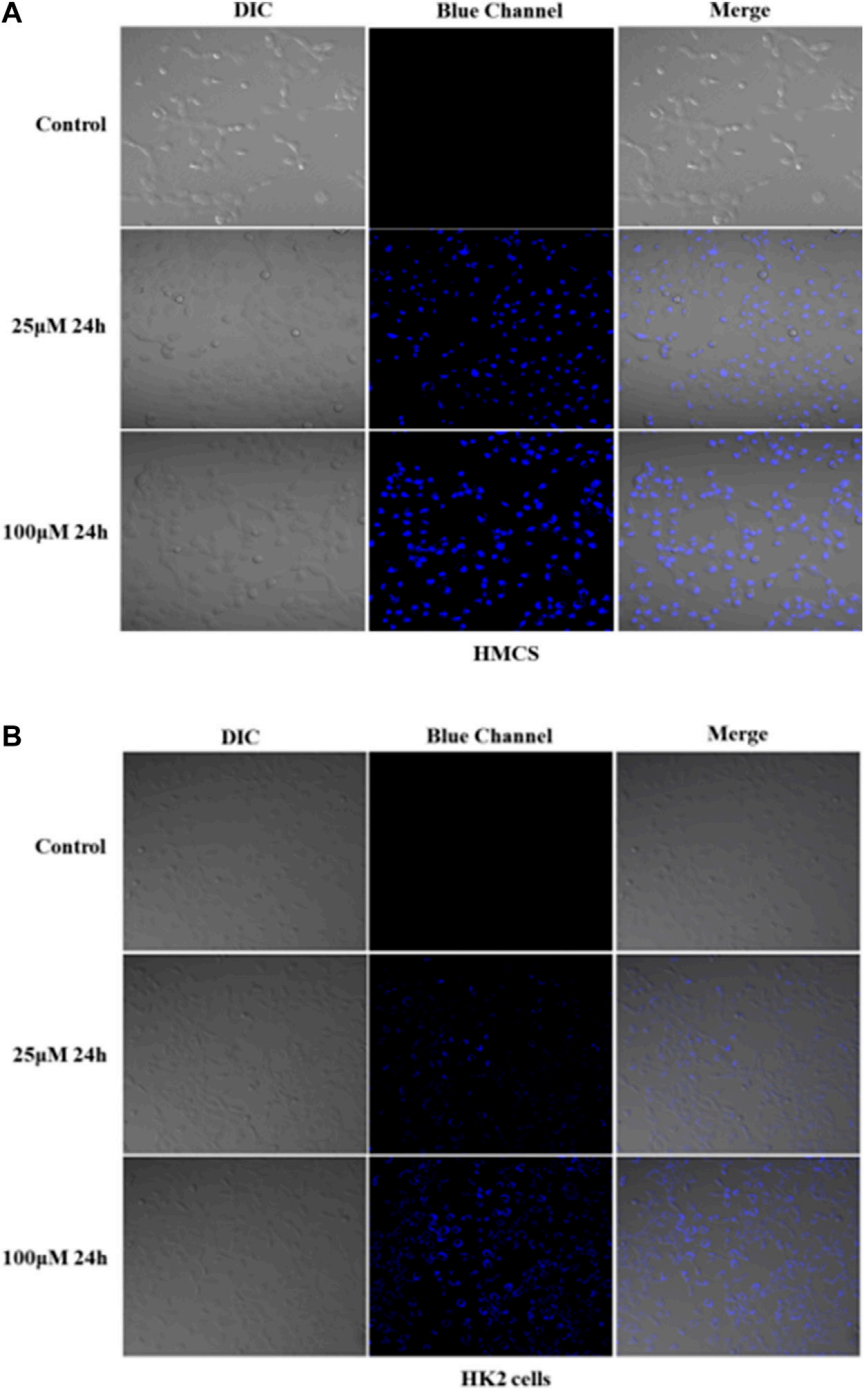
Confocal images of live HMCs **(A)** and HK2 cells **(B)** incubated with 25 and 100 μM Q [8]@PEG-APTS for 24 h (more details can be seen in Supplementary Figures S7, S8). Blue channel: λ_ex_ = 405 nm, λ_em_ = 450 nm. DIC, differential interference contrast in transmitted light images.

Because of the good absorption for cells, Q [8]@PEG-APTS may be a potential biochemical tool to achieve *in vivo* cell imaging. Moreover, interestingly, uneven distribution patterns of Q [8]@PEG-APTS within HMCs and HK2 cells were observed. Blue fluorescence dots clustered around the perinuclear of HMCs were observed while diffused over the entire cytoplasm in HK2 cells, and cells exhibited elevated fluorescence intensity with the increase in culture concentration (Figure 5, Supplementary Figures S7, S8). In this context, Rajdeep Chowdhury et al. found that the fluorescent probe, 8-hydroxy-pyrene-1,3,6-trisulfonate (HPTS), could be predominantly localized in the lysosome of lung cancer cells (Chowdhury et al., 2015). However, other researchers observed HPTS diffused in the cytosol of Hela cells and Chinese hamster ovary cells (Sen Mojumdar et al., 2013; Cao et al., 2021). Therefore, the different distribution of Q [8]@PEG-APTS in these two types of renal residual cells has been the prime novelty of this study and this property may be helpful to identify diverse cell types when tracing live cells in the kidney. However, there are still many foundations works to apply Q [8]@PEG-APTS for the detection and diagnosis of kidney disease in clinical.

## Experimental

Materials; instruments; and advanced characterization techniques, such as ^1^H NMR, FT-IR, fluorescence spectrum, *etc.*, are detailed in the supporting information. The detailed synthesis of PEG-APTS (Supplementary Scheme S2) is detailed in the supporting information. Herein, PEG-Cl (4 g, 2 mmol), 8-hydroxy-1,3,6-pyrene trisodium (1.048 g, 2 mmol), and 8.28 g anhydrous K_2_CO_3_ were added to 150 ml anhydrous DMF, followed by heating and refluxing at 130°C for 48 h. In the end, the solvent was removed by vacuum evaporation to obtain 2.8 g of brick-red solid crude product, of which 1.6 g of a brick-red solid product was obtained after purification by column chromatography using methanol as eluent. Detailed characterizations are given in the supporting information.

## Conclusion

In this study, supramolecular fluorescent biomaterials Q [8]@PEG-APTS have been constructed by using synthesized polymers PEG-APTS and Q [8] through outer surface interaction. The biomaterials have been confirmed to possess a stable and uniform structure with brilliant fluorescence properties. Most importantly, the introduction of the positive density on the outer surface of Q [8]@PEG-APTS has made it faster to go through the glomerular filtration barrier and enter the kidney resident cells. Subsequently, this biomaterial has been able to accumulate selectively in the cytoplasm and nucleus of HK2 and HMCs cells, respectively, within 24 h, indicating that Q [8]@PEG-APTS may help in identifying different cell types when tracking live cells in the kidney. Follow-up, studies on the different recognition mechanisms of Q [8]@PEG-APTS in tracking live kidney cells are important to facilitate the simultaneous imaging and identification of mixed kidney cells, which is more suitable for early and rapid diagnosis of AKI, providing a theoretical basis for the potential application of Q [*n*]-based biomaterials in kidney research and therapy.

## Data Availability

The original contributions presented in the study are included in the article/Supplementary Material, further inquiries can be directed to the corresponding authors.
