# Supplementary material for: Cucurbit [8] uril-based supramolecular fluorescent biomaterials for cytotoxicity and imaging studies of kidney cells
Source: Front Chem. 2022 Aug 24;10:974607. doi: 10.3389/fchem.2022.974607 (PMC9451006; doi:10.3389/fchem.2022.974607)
Supplement: Supplementary file 1 [file DataSheet1.docx]

**Supporting Information**

**Cucurbit[8]uril-based Supramolecular Fluorescent Biomaterials for Cytotoxicity and Imaging Studies of Kidney Cells**

Han Xiao ^a^†, Xia Yang ^a^†, Li Yang ^a^, Dan Yang ^b^, Yang Luo ^b^, Hai-ping Yang **^*^**^a^, Zhu Tao ^b^, Xin Xiao^b^, and Qiu Li **^*^**^a^

^a^ Department of Nephrology, Children’s Hospital of Chongqing Medical University, National Clinical Research Center for Child Health and Disorders, Ministry of Education Key Laboratory of Child Development and Disorders, Chongqing Key Laboratory of Pediatrics, Chongqing 400014, China

^b^ Key Laboratory of Macrocyclic and Supramolecular Chemistry of Guizhou Province, Guizhou University, Guiyang 550025, China.

Content

[**1.** **Materials** 2](#_Toc107752255)

[**2.** **Instruments** 2](#_Toc107752256)

[**3.** **Synthesis** 3](#_Toc107752257)

[**3.1. Synthesis of PEG-Cl** 3](#_Toc107752258)

[**Scheme S1.** Organic synthesis route design of **PEG-Cl**. 3](#_Toc107752259)

[**3.2. Synthesis of PEG-APTS** 4](#_Toc107752260)

[**Scheme S2.** Organic synthesis route design of **PEG-APTS**. 4](#_Toc107752261)

[**4.** **Experimental method** 4](#_Toc107752262)

[**4.1 ^1^H NMR measurements** 4](#_Toc107752263)

[**Figure S1.** ^1^ H NMR spectrum (400 MHz, D_2_O, room temperature) of **PEG-APTS**. 5](#_Toc107752264)

[**4.2 Ultraviolet-visible spectrophotometry measurements** 6](#_Toc107752265)

[**Figure S2.** UV–vis spectra of **PEG-APTS** (20 μM) in the presence of various Q[8] concentrations. 6](#_Toc107752266)

[**4.3 Fluorescence emission spectrum measurements** 7](#_Toc107752267)

[**Figure S3.** Fluorescence emission spectra of **PEG-APTS** (20 μM) in the presence of various Q[8] concentrations. 7](#_Toc107752268)

[**Figure S4.** The DLS data of 6 μM Q[8], 3 μM PEG-APTS, and Q[8]@PEG-APTS (6 μM Q[8], 3 μM PEG-APTS). 8](#_Toc107752269)

[**Figure S5.** The SEM images of Q[8]@PEG-APTS. 8](#_Toc107752270)

[**4.4 Fourier transform infrared spectroscopy (FT-IR) measurements** 9](#_Toc107752271)

[**Figure S6.** The FT-IR spectroscopy **Q[8]@PEG-APTS**, **PEG-APTS**. 9](#_Toc107752272)

[**4.5 Dynamic light scattering (DLS) measurements** 10](#_Toc107752273)

[**4.6 Scanning electron microscope (SEM) observations** 10](#_Toc107752274)

[**4.7 Cell treatment and images** 10](#_Toc107752275)

[**Figure S7.** Confocal images of live HMCs incubated with 25μM and 100μM **Q[8]@PEG-APTS** for 1h and 24h. Blue channel: λ_ex_ = 405 nm, λ_em_ =450 nm. DIC: differential interference contrast in transmitted light images. 11](#_Toc107752276)

[**Figure S8.** Confocal images of live HK2 cells(d) incubated with 25μM and 100μM **Q[8]@PEG-APTS** for 1h and 24h. Blue channel: λ_ex_ = 405 nm, λ_em_ =450 nm. DIC: differential interference contrast in transmitted light images. 12](#_Toc107752277)

[**4.8 Cell Counting Kit-8 (CCK-8)** 13](#_Toc107752278)

[**4.9 Flow cytometry assay** 13](#_Toc107752279)

1. **Materials**

Methoxy polyethylene glycol (PEG-OH, M_n_ = 2000), SOCl_2_, 8-hydroxy-1,3, 6-trisulfonic pyrene (APTS), toluene, methylene chloride, anhydrous sodium sulfate, ether, deuterium dimethyl sulfoxide, deuterium oxide, methyl alcohol, DMF and anhydrous potassium carbonate were purchased from Aladdin (Shanghai, China). All the solvents used below are purchased from Aladdin (Shanghai, China). All reagents were of analytical reagent grade and were used without further purification. Deionized water was used throughout. Q[8] was synthesized by our laboratory.

1. **Instruments**

Varian Cary Eclipse Fluorescence emission spectra (Varian, Inc., Palo Alto, CA, USA). JEOL JNM-ECZ400s ^1^H NMR spectra. 8453 UV-visible spectra from Agilent (Agilent Technologies, Santa Clara, CA, USA). Zeiss Sigma VP field emission scanning electron microscope (Germany). Brookhaven BI-200SM laser light scattering spectrometer.

1. **Synthesis**

**3.1.** **Synthesis of PEG-Cl**

**Scheme S1.** Organic synthesis route design of **PEG-Cl**.

30 g methoxy polyethylene glycol (2000) was weighed into a 250 mL round-bottom flask, and 15 mL SOCl_2_ was added to the dropping funnel. Added 40 mL toluene to the round-bottom flask and the dropping funnel for dissolution and dilution respectively. The diluted SOCl_2_ solution was added drop by drop into a round-bottom flask at room temperature and then heated to 90 ℃ for refluxed 20 h after the dropwise addition. The reaction mixture was concentrated to remove toluene at the end of the reaction, and added 100 mL of deionized water to it, stirring until it was completely dissolved. The aqueous solution was extracted with methylene chloride, then the organic phase was dried and concentrated with K_2_CO_3_. After concentration to about 20 mL, the organic phase was dropped into 500 mL diethyl ether to precipitate the product. After filtering, the product was placed in a vacuum drying oven until constant weight, and the product was 21.4 g with a yield of 71.3%. There is no peak at 4.56 ppm in the NMR hydrogen spectrum of deuterium dimethyl sulfoxide as the solvent, indicating that the hydroxyl groups at the end of methoxyethylene glycol are all replaced by chlorine.

**3.2. Synthesis of PEG-APTS**

**Scheme S2.** Organic synthesis route design of **PEG-APTS**.

The products synthesized in the previous step, methoxy polyethylene glycol chloride 4 g (2 mmol) and 8-hydroxy-1,3,6-pyrene trisodium 1.048 g (2 mmol), were weighed into a 250 mL round-bottom flask, in which 8.28 g anhydrous K2CO3 was added, and 150 mL anhydrous DMF was added, then heated to 130 ℃ and reacted for 48 hours at circulation reflux. After the reaction, the filtrate was concentrated on a rotary evaporator to remove DMF in the reaction and obtained 2.8 g of brick red solid. Methanol was used as the developing agent; the brick red solid was analyzed by chromatography to obtain 1.6 g of the product.

1. **Experimental method**

**4.1** **^1^H NMR measurements**

^1^H NMR spectra were measured on a JEOL JNM-ECZ 400s ^1^H NMR spectrometer at 298 K, using D_2_O as a field frequency lock. The observed chemical shift was one part per million (ppm) relative to the built-in tetramethylsilane (TMS) standard (0.0 ppm).

These results as shown in Figure S1, it is evident that specific proton of PEG-APTS, namely Ha, Hb, Hc, and Hd shifts δ (ppm): 3.17(-CH_3_), 3.48 (-OCH_2_CH_2_O), 8.21-8.25 (phenyl), 8.68-8.71 (phenyl), 8.78-8.80 (phenyl), 8.94-8.99 (phenyl), 8.87-8.89 (phenyl).


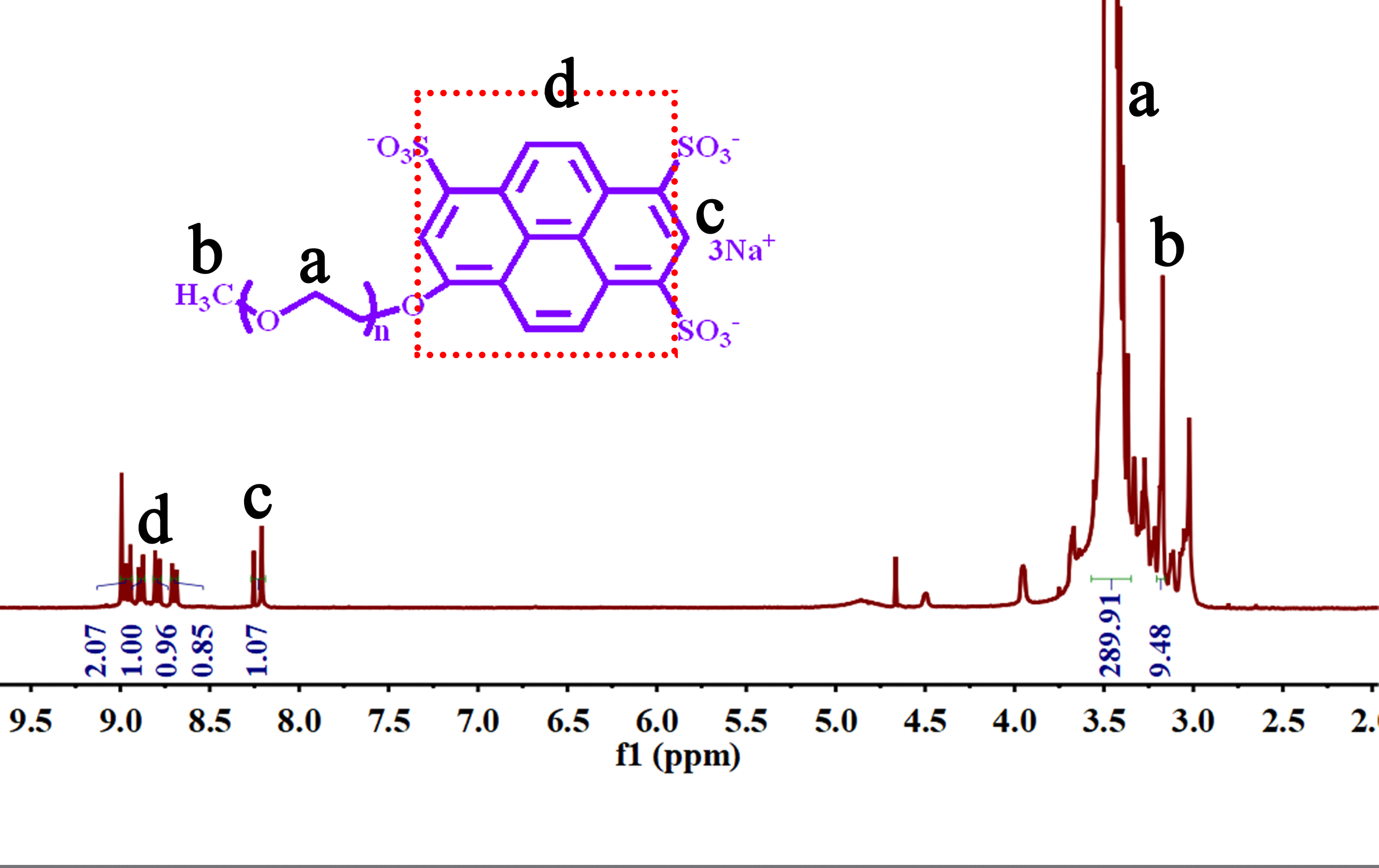


**Figure S1.** ^1^ H NMR spectrum (400 MHz, D_2_O, room temperature) of **PEG-APTS**.

**4.2** **Ultraviolet-visible spectrophotometry measurements**

Ultraviolet-visible spectrophotometry was measured on 8453 UV-visible spectra from Agilent (Agilent Technologies, Santa Clara, CA, USA) spectrometer at 298 K. All solutions were filled in 10 mm path length cells, and the scanning wavelength ranged from 200 nm to 800 nm at a scan rate of 400 nm per min.

The mole ratio method was used to detect the interaction mode between Q[8] and PEG-APTS. The specific method was as follows: add 0, 0.2, 0.4 … 4.5 times Q[8] in the fixed PEG-APTS (60 μM), the fixed concentration was 20 μM, at 298 K. obtained UV-vis absorption spectra.

**Figure S2.** UV–vis spectra of **PEG-APTS** (20 μM) in the presence of various Q[8] concentrations.

**4.3** **Fluorescence emission spectrum measurements**

Fluorescence spectra measurements were performed on a Varian Cary Eclipse Fluorescence emission spectra (Varian, Inc., Palo Alto, CA, USA) spectrometer at 298 K. All solutions were filled in 10 mm path length quartzose cuvette, and the scanning wavelength ranged from 350 nm to 550 nm. Fluorescence spectrometry conditions: excitation wavelength of 392 nm, slit width of 5 nm, a voltage of 400 V.

**Figure S3.** Fluorescence emission spectra of **PEG-APTS** (20 μM) in the presence of various Q[8] concentrations.


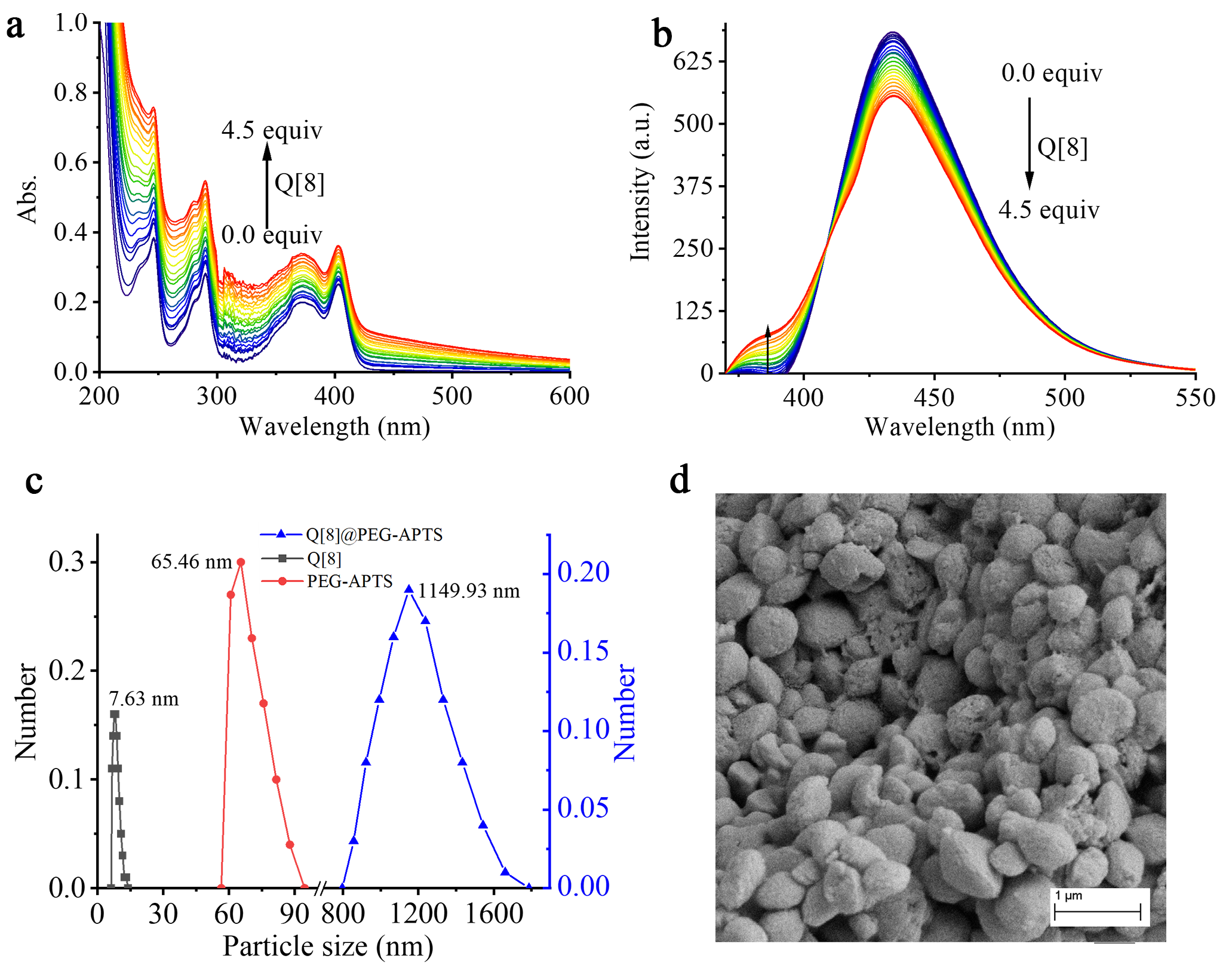


**Figure S4.** The DLS data of 6 μM Q[8], 3 μM PEG-APTS, and Q[8]@PEG-APTS (6 μM Q[8], 3 μM PEG-APTS).


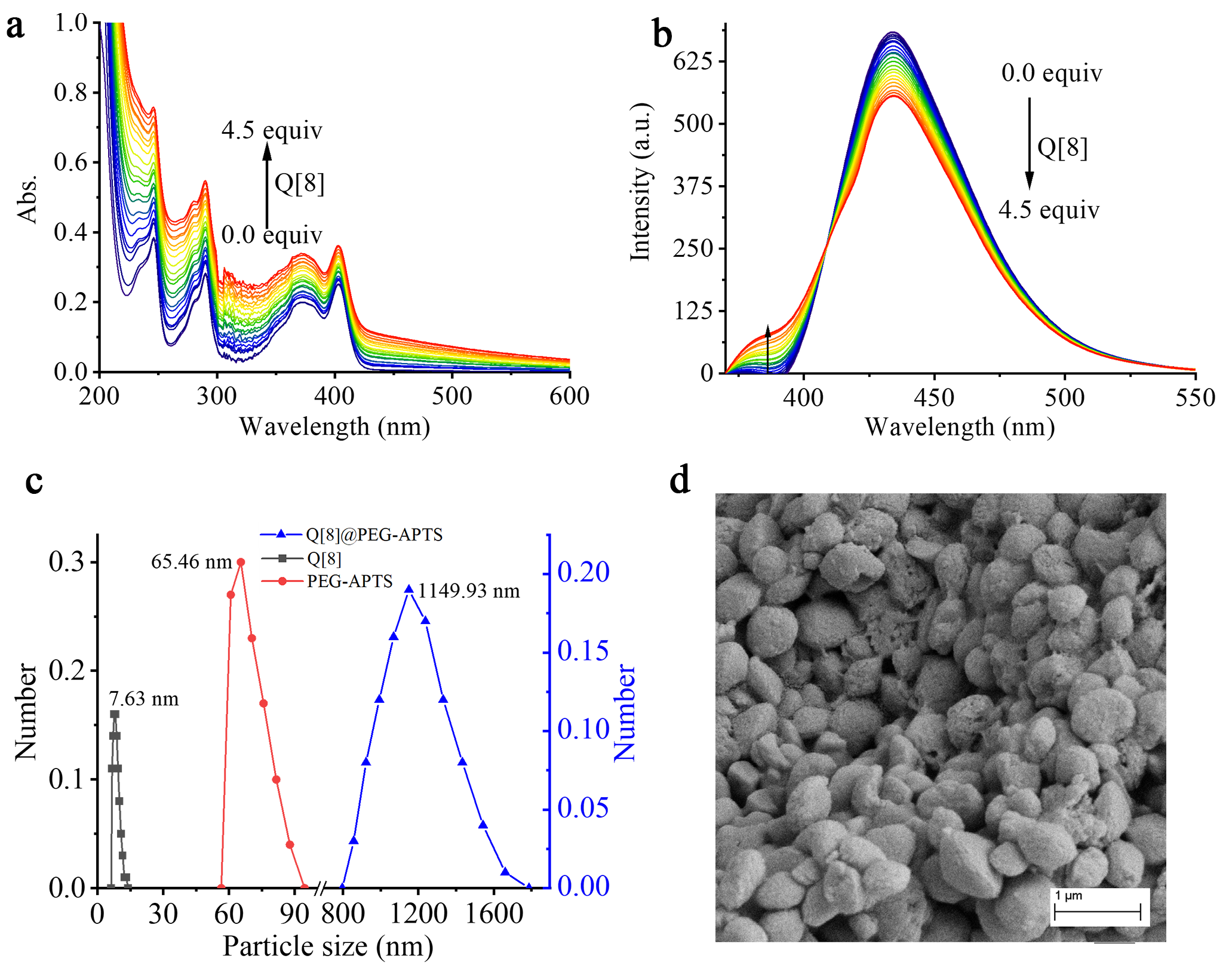


**Figure S5.** The SEM images of Q[8]@PEG-APTS.

**4.4 Fourier transform infrared spectroscopy (****FT-IR)** **measurements**

PEG-APTS (20 mg) and Q[8] @PEG-APTS (molar ratio = 1:2, 20 mg) were dried in the oven and ground in an agate mortar to powder.  Dry KBr (spectral pure) was added to the sample and mixed evenly. The sample was pressed under 20 MPa pressure using a tablet clamp for infrared spectral analysis.


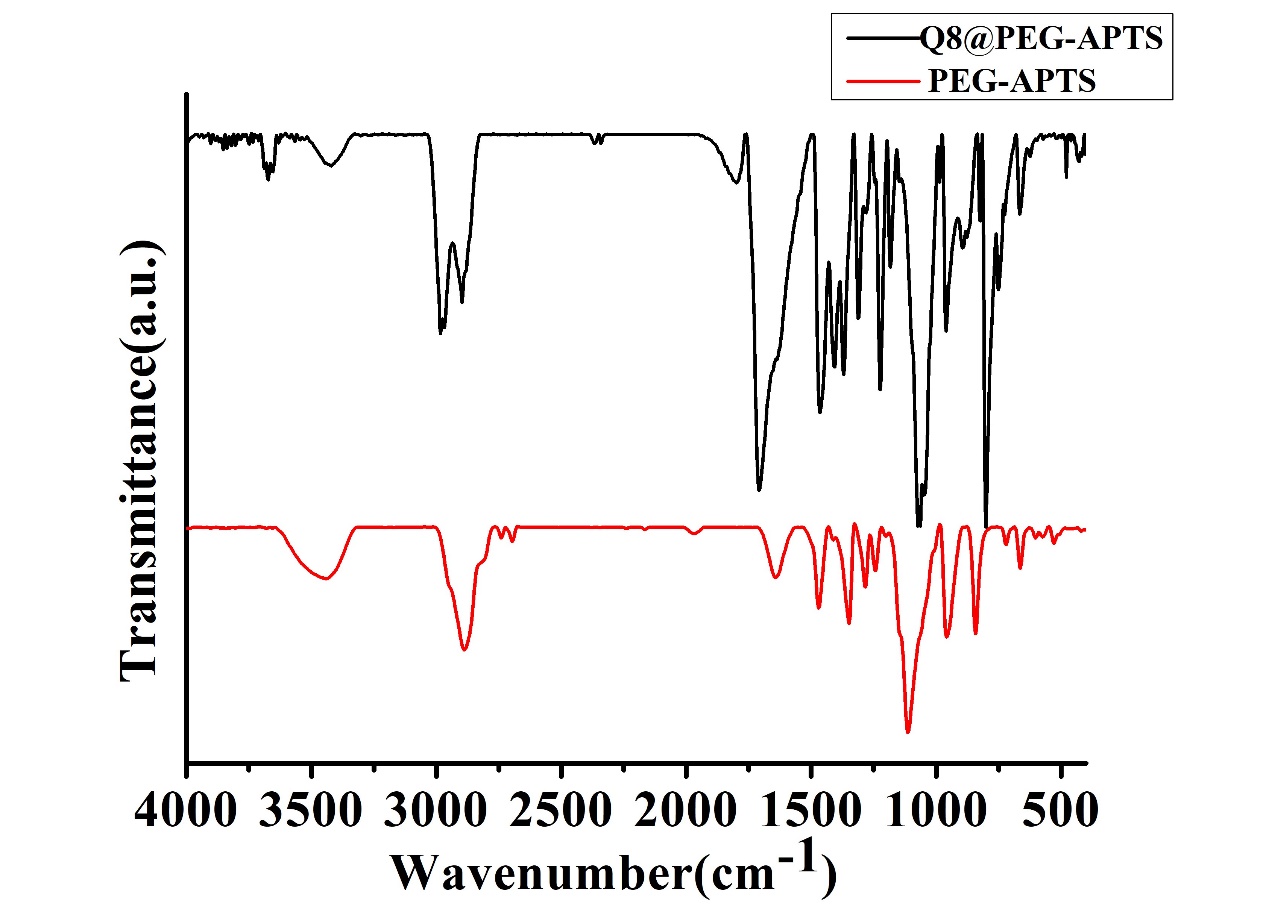


**Figure S6.** The FT-IR spectroscopy **Q[8]@PEG-APTS**, **PEG-APTS**.

**4.5 Dynamic light scattering (DLS) measurements**

Dynamic light scattering experiments were carried out on the Brookhaven BI-200SM laser light scattering spectrometer. The sample was filtered through the PES 13mm* 0.22 μm filter before loading. The solution was placed in a plurality of path length plastic test tubes in 1 cm and measured at 90° at 298 K.

**4.6 Scanning electron microscope (****SEM) observations**

10 μL aqueous solution of Q[8]@PEG-APTS (molar ratio = 4.5:1, 60 μM) is added onto silicon wafers, then the wafers were placed in a cool and ventilated place to dry. The sample was coated with platinum before SEM characterization.

**4.7 Cell treatment and images**

Human mesangial cells (HMCs) were grown in 1640 medium containing 10% FBS，1‰ insulin-transferrin-sodium selenite (ITS), 100 U / mL penicillin, and streptomycin. Human proximal tubular epithelial cell lines (HK2 cells) were grown in a DMEM-F12 medium containing 10% FBS, 100 U/mL penicillin, and streptomycin. Both cell types were cultured in the medium with different concentrations of Q[8]@PEG-APTS in a humidified atmosphere with 5% CO_2_ at 37°C for 0h, 1h,6h,12h, and 24h respectively. Before detection, the culture medium was removed and the fresh one was added. Live cells were captured under blue and DIC channels with a confocal microscope.


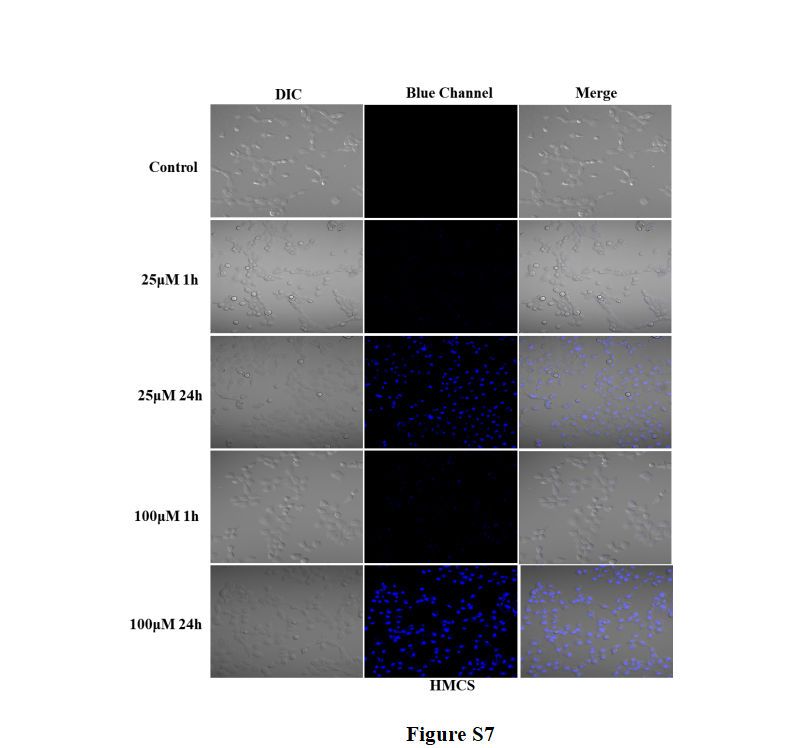


**Figure S7.** Confocal images of live HMCs incubated with 25μM and 100μM **Q[8]@PEG-APTS** for 1h and 24h. Blue channel: λ_ex_ = 405 nm, λ_em_ =450 nm. DIC: differential interference contrast in transmitted light images.


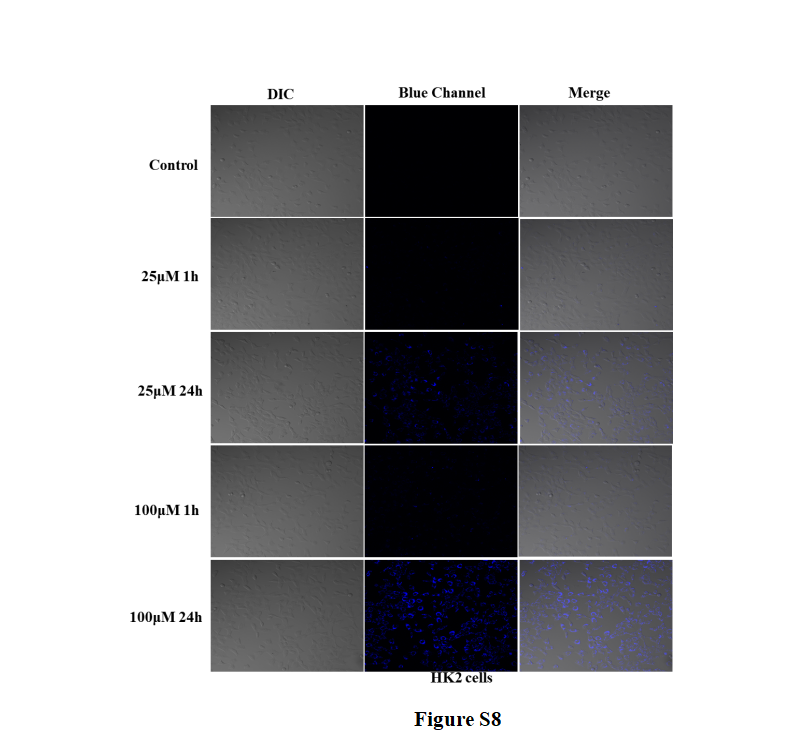


**Figure S8.** Confocal images of live HK2 cells(d) incubated with 25μM and 100μM **Q[8]@PEG-APTS** for 1h and 24h. Blue channel: λ_ex_ = 405 nm, λ_em_ =450 nm. DIC: differential interference contrast in transmitted light images.

**4.8 Cell Counting Kit-8 (CCK-8)**

Cells were cultured in 96‐well microplates with 100μL cell medium at a density of 2 × 10^4^/well. After the incubation of gradient concentration and time, cells were washed twice with PBS buffer and replaced with a fresh medium. Then 10 μL of CCK-8 reagent (Abclonal, Wuhan, China) was added to each well. The plates were placed in 37 °C incubators for 2h and then measured the absorbance at 450 nm wavelength with a microplate reader (Bio‐Rad, Hercules, CA, USA).

**4.9 Flow cytometry assay**

Following treatment with Q[8]@PEG-APTS, cells were harvested by trypsinization and washed with PBS buffer. 500μL binding buffer was added to resuspend cells. Then each group of cells was stained with 5μL annexin V-APC and 5μL 7-AAD for 15min at room temperature (Keygene Biotech, Jiangsu, China). The apoptotic rates and the mean fluorescence intensity (MIF) of Q[8]@PEG-APTS absorbed by cells were detected with the flow cytometer.
